# Frequent lucid dreaming associated with increased functional connectivity between frontopolar cortex and temporoparietal association areas

**DOI:** 10.1038/s41598-018-36190-w

**Published:** 2018-12-12

**Authors:** Benjamin Baird, Anna Castelnovo, Olivia Gosseries, Giulio Tononi

**Affiliations:** 1 Wisconsin Institute for Sleep and Consciousness Department of Psychiatry, University of Wisconsin, Madison, USA; 2 Sleep and Epilepsy Center Neurocenter of Southern Switzerland, Civic Hospital (EOC) of Lugano, Lugano, Switzerland; 3 Coma Science Group GIGA-Consciousness, University of Liege, Liege, Belgium

## Abstract

Humans typically lack awareness that they are dreaming while dreaming. However, at times a remarkable exception occurs and reflective consciousness can be regained while dreaming, referred to as lucid dreaming. While most individuals experience lucid dreams rarely there is substantial variance in lucid dream frequency. The neurobiological basis of lucid dreaming is unknown, but evidence points to involvement of anterior prefrontal cortex (aPFC) and parietal cortex. This study evaluated the neuroanatomical/neurofunctional correlates of frequent lucid dreams and specifically whether functional connectivity of aPFC is associated with frequent lucid dreams. We analyzed structural and functional magnetic resonance imaging from an exceptional sample of fourteen individuals who reported ≥3 lucid dreams/week and a control group matched on age, gender and dream recall that reported ≤1 lucid dream/year. Compared to controls, the frequent lucid dream group showed significantly increased resting-state functional connectivity between left aPFC and bilateral angular gyrus, bilateral middle temporal gyrus and right inferior frontal gyrus, and higher node degree and strength in left aPFC. In contrast, no significant differences in brain structure were observed. Our results suggest that frequent lucid dreaming is associated with increased functional connectivity between aPFC and temporoparietal association areas, regions normally deactivated during sleep.

## Introduction

For reasons not currently understood, humans are typically unaware that they are dreaming while dreaming. At times, however, a remarkable exception occurs and we can become aware of the fact that we are dreaming, a state referred to as lucid dreaming^1^. During lucid dreams, one becomes aware that one is dreaming while remaining physiologically asleep and immersed within a dream environment that often appears strikingly realistic. In addition to the metacognitive awareness of one’s state of consciousness, during lucid dreams it is also common to regain episodic memory for waking life as well as the ability to volitionally control actions within the dream (e.g.^2,3^). Despite initial skepticism from some scientists and philosophers, lucid dreaming has been demonstrated to be objectively verifiable through volitional eye movement signals which can be recorded in the electrooculogram during polysomnography-verified REM sleep^4^ (for replications and extensions see, e.g., refs.^5–7^; for recent implementations see, e.g., refs.^8–10^). For most individuals lucid dreams spontaneously occur infrequently, however there is substantial variation in lucid dream frequency, ranging, by current estimates, from never (approximately 40–50%) to monthly (approximately 20%) to a small percentage of people that experience lucid dreams several times per week or in some cases every night^11,12^. This variation invites the question of whether the frequency of lucid dreams is related to individual differences in anatomical or functional properties of the brain.

The prefrontal cortex (particularly the lateral and rostrolateral regions), parietal cortex and lateral middle temporal cortex show low regional cerebral blow flow (rCBF) throughout sleep, including during REM sleep^13–15^, the stage of sleep most strongly associated with dreaming. Hypoactivity of these regions has been postulated to underlie the diminished self-awareness and volitional control during dreaming^15,16^. Consistent with this, a functional magnetic resonance imaging (fMRI) case study found increased BOLD signal in many of these same regions during lucid compared to non-lucid REM sleep, including the anterior prefrontal cortex (aPFC), bilateral inferior parietal lobule (IPL), precuneus and inferior/middle temporal gyrus (ITG/MTG)^9^. However, these results should be interpreted cautiously given that they are derived from a single subject, and no group-level fMRI study of lucid REM sleep has yet been undertaken. EEG studies have also reported increased activity in the beta band over parietal regions^17^ or gamma band in frontal regions^18^ during lucid compared to baseline REM sleep. However, overall EEG studies of lucid dreaming show considerable discrepancies and at the current time these results should be interpreted cautiously given methodological issues such as low statistical power^19,20^.

Despite these caveats, evidence linking frontopolar and parietal regions to lucid dreaming is consistent with the role of these regions in metacognitive functions. Across the literature, a convergence of evidence indicates that aPFC in particular is a critical part of the neuroanatomical basis of metacognitive processes. For example, research has found that aPFC shows increased activation during self-reflection on internal states, such as the evaluation of one’s own thoughts and feelings^21,22^. Individuals can also learn to voluntarily modulate activity in aPFC through a metacognitive awareness strategy^21^. Furthermore, inter-individual variance in metacognitive ability has also been linked to aPFC gray matter volume^23,24^ and aPFC functional connectivity^24^. Finally, patients with damage to this region frequently display metacognitive deficits such as an inability to monitor disease symptoms or accurately appraise their cognitive functioning^25,26^, similar to the lack of metacognitive insight into the global state of consciousness characteristic of non-lucid REM sleep dreams^27^.

As the initiation of lucid dreaming requires one to achieve metacognitive awareness of the state of consciousness one is in, these findings motivate the hypothesis that individual differences in the anatomy or functional connectivity of aPFC could be associated with the frequency of lucid dreams. Indeed, lucid dreaming presents a unique experimental paradigm to further explore the link between aPFC and metacognitive awareness^28,29^. In further support of a connection between the metacognitive functions of aPFC and lucid dreaming, a recent study found increased gray matter volume in two regions of the frontal pole in individuals who scored higher on a scale assessing the frequency of lucid dreams and/or dream content hypothesized to be related to lucidity^30^. Additionally, these same regions also showed increased BOLD activation in the monitoring component of a metacognitive thought-monitoring task. However, a limitation of the study was a lack of specific assessment of lucid dream frequency in the “high lucidity” and “low lucidity” groups (lucid dream frequency for the two groups was not reported). Furthermore, the groups were distinguished based on a median split on scores to a composite measure that also included elements that may have varied with dream recall frequency, making it unclear whether the results could have been partly influenced by differences in dream recall. In summary, research points to the possibility that frontoparietal cortex, and aPFC in particular, could be associated with lucid dream frequency. However, an analysis of brain structure and function in individuals who experience frequent lucid dreams, while also controlling for dream recall frequency, is needed.

In the current research we evaluated an exceptional sample of individuals who reported lucid dreams spontaneously in the range of approximately every other night to multiple times per night compared to a control group matched on age, gender and dream recall frequency but who reported lucid dreams once per year or less. The primary aim of the study was to test whether differences in brain structure and/or functional connectivity are associated with frequent lucid dreams while also controlling for dream recall frequency. Based on the research reviewed above, our primary analysis investigated whether individuals who have frequent lucid dreams would show increased gray matter density and/or resting-state functional connectivity of aPFC. For analysis of structural data, we first employed a whole-brain voxel-based morphometry (VBM) analysis^31^, followed by a region-of-interest (ROI) analysis of the aPFC regions reported to be associated with lucid dream frequency in a previous study^30^. For resting-state functional connectivity (rsfcMRI) analysis, we employed seed-based whole-brain functional connectivity analysis of aPFC, based on the aPFC activation peak reported in the fMRI case study of lucid REM sleep^9^, which allowed us to explore differences in aPFC functional connectivity with all other brain regions between groups. We additionally employed a follow-up whole-brain graph-theoretic analysis to examine differences in functional network properties across all brain areas between groups in a data-driven approach, as well as evaluated differences in within-network and between-network connectivity in large-scale resting-state networks (LSNs)^32^. Finally, we evaluated several additional cognitive variables which have been hypothesized to be associated with lucid dreaming and have been linked to PFC function, including working memory capacity, trait mindfulness and prospective memory (e.g., refs.^2,33,34^), in order to test for between-group differences and, if necessary, to be able to control for these variables in our MRI analysis.

## Results

### Demographic and behavioral results

The mean age for both groups was 22.6 ± 5.4 [M ± SD] (range = 18–34) and both groups were composed of 5 males and 9 females. There was no significant difference in dream recall between the control group (median = 5–6 per week; IQR = 2) and lucid dream group (median = 7 per week; IQR = 1) [*Z* = 1.70, *p* = 0.11, Mann-Whitney U-test; see *Methods* for details on dream recall case-control matching]. All 28 participants reported high dream recall (≥3–4 per week). The frequent lucid dream group reported significantly more lucid dreams (median = 5–6 per week; IQR = 1) compared to the control group (median = 0 per week; IQR = 0) [*Z* = 4.68, *p* < 10^−6^, Mann-Whitney U-test]. The frequent lucid dream group reported a median of 75 lucid dreams in the last 6 months, a median of 90 lucid dreams for the highest number of lucid dreams in any 6-month period, and reported experiencing lucid dreams on average for 9.5 ± 5.8 [M ± SD] years. No significant differences between groups were observed for working memory capacity (OSpan, RotSpan, SymSpan), or questionnaire assessments of mind-wandering frequency, prospective or retrospective memory or trait mindfulness (all *p* ≥ 0.25, two-tailed independent samples *t*-test; Table 1).

**Table 1:** Demographic, behavioral and questionnaire data for the frequent lucid dream group and control group.

		Lucid dream group	Control group	*Z*	*p*
		(*N* = 14)	(*N* = 14)		
Dream recall	Dream recall (median)	7 per week	5–6 per week	1.70	0.11
	Lucid dreams (median)	5–6 per week	0 (no lucid dreams)	4.68	<10^−6^
	Lucid dreams last 6 mo (median)	75			
	Lucid dreams most 6 mo (median)	90			
		***M*** **(SD)**	***M*** **(SD)**	***t*** **(26)**	***p***
Demographic data	Gender (Female|Male)	9|5	9|5		
	Age	22.64 (5.46)	22.66 (5.47)	−0.01	0.99
Working memory	OSpan	32.00 (9.57)	33.21 (12.15)	−0.29	0.77
	RotSpan	12.29 (6.37)	14.14 (5.96)	−0.80	0.43
	SymSpan	16.07 (7.78)	12.93 (6.22)	1.18	0.25
Questionnaires	IPI Mind-wandering	3.36 (0.85)	3.32 (0.95)	0.12	0.90
	PRMQ Retrospective	3.46 (0.69)	3.71 (0.65)	−0.98	0.34
	PRMQ Prospective	3.02 (0.61)	3.36 (0.77)	−1.32	0.19
	TMS Decentering	13.36 (3.50)	11.93 (3.36)	1.10	0.28
	TMS Curiosity	18.50 (4.26)	15.36 (4.41)	1.92	0.07

*Note*. OSpan = Operation Span, SymSpan = Symmetry Span, RotSpan = Rotation Span, IPI = Imaginal Process Inventory, PRMQ = Prospective and retrospective memory questionnaire, TMS = Toronto Mindfulness Scale.

### Voxel-based morphometry (VBM)

No suprathreshold clusters were observed for either the frequent lucid dream group contrasted with the control group or the control group contrasted with the frequent lucid dream group at the whole brain level either for raw gray matter density values or after proportional scaling gray matter values by total intracranial volume (all *p* > 0.05, two-tailed independent samples *t*-test, corrected for multiple comparisons at the cluster level). No significant differences in gray matter density were observed for ROIs in left prefrontal cortex (*t*(26) = −0.47, *p* = 0.65, two-tailed independent samples *t*-test), right prefrontal cortex (*t*(26) = −0.36, *p* = 0.72, two-tailed independent samples *t*-test), or the left (*t*(26) = −0.40, *p* = 0.69, two-tailed independent samples *t*-test) or right (*t*(26) = −1.31, *p* = 0.20, two-tailed independent samples *t*-test) hippocampus based on the regions reported in ref.^30^. Total hippocampal volume (extracted from FreeSurfer segmentation) also showed no significant differences between groups for either left (*t*(26) = 0.14, *p* = 0.89, two-tailed independent samples *t*-test) or right (*t*(26) = 0.32, *p* = 0.75, two-tailed independent samples *t*-test) hippocampus.

### Seed-based whole-brain resting-state functional connectivity

**Figure 1:**
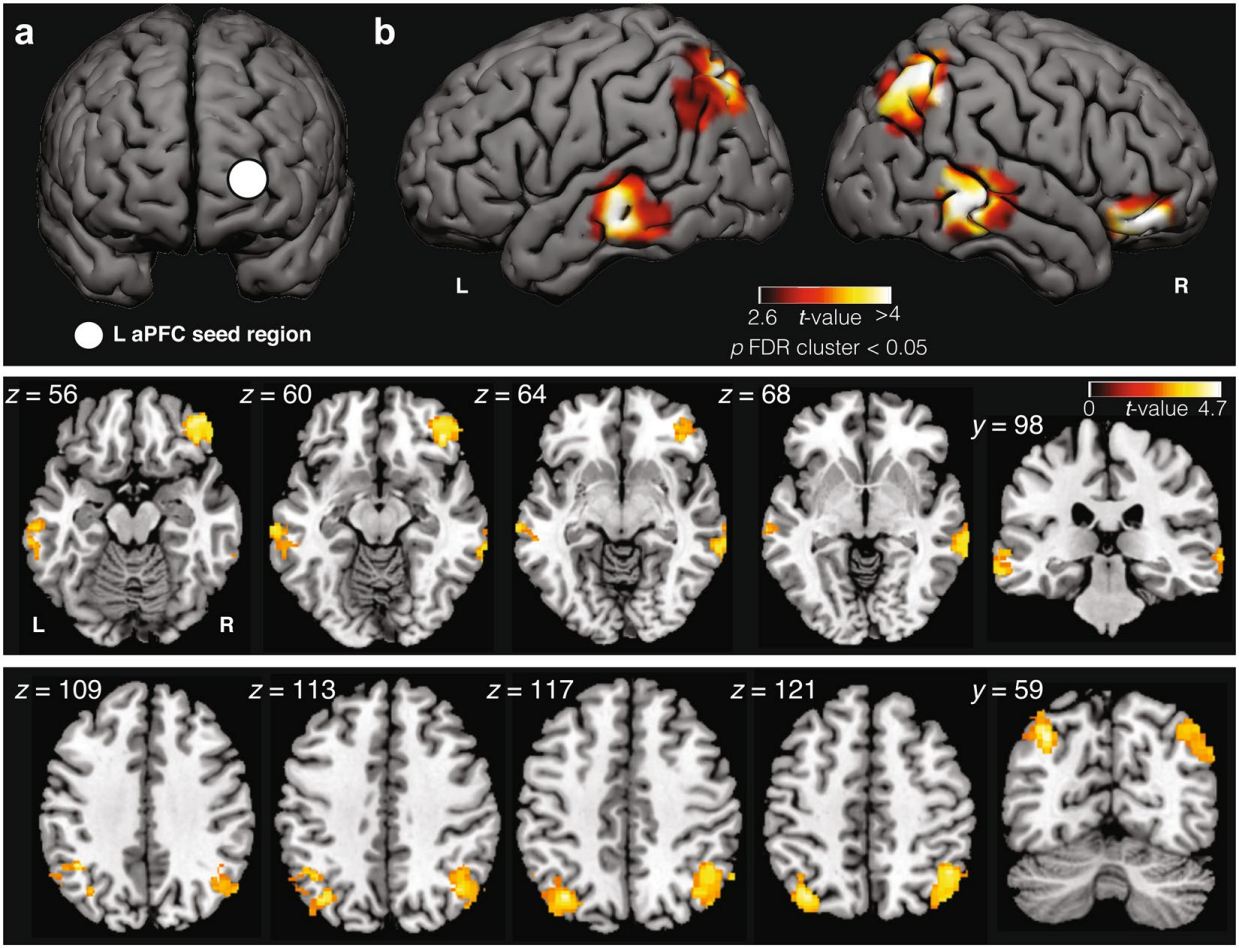
Seed-based resting-state functional connectivity differences between frequent lucid dream and control groups. Top panel: (**a**) Seed region of left aPFC with significant differences between groups. To estimate connectivity, spherical ROIs of 6 mm radius were defined in aPFC based on the peak voxel reported in Dresler *et al*.^9^ which had increased fMRI BOLD signal response during signal-verified lucid REM sleep dreaming. **(b)** The frequent lucid dream group showed increased resting-state functional connectivity between left aPFC and the bilateral angular gyrus (AG), bilateral middle temporal gyrus (MTG) and right inferior frontal gyrus (IFG). All clusters are significant at *p* < 0.05, corrected for multiple comparisons at the cluster level. Middle panel: Volume slices illustrating bilateral MTG and IFG results. Bottom panel: Volume slices illustrating bilateral AG results.

There were no significant differences in in-scanner head motion (mean framewise displacement) between the frequent lucid dream group (*M* = 0.07, *SD* = 0.03) and control group (*M* = 0.07, *SD* = 0.04) (*t*(26) = 0.72, *p* = 0.48, two-tailed independent samples *t*-test). As shown in Fig. 1 and Table 2, compared to the control group, the frequent lucid dream group showed significantly increased functional connectivity between left aPFC and five clusters: the left and right inferior parietal lobule (IPL), left and right middle temporal gyrus (MTG) and right inferior frontal gyrus (IFG) (all *p* < 0.05, two-tailed independent samples *t*-test, corrected for multiple comparisons at the cluster level; Table 2). The frequent lucid dream group also displayed reduced functional connectivity between left aPFC and the bilateral insula (all *p* < 0.05, two-tailed independent samples *t*-test, corrected for multiple comparisons at the cluster level; Table 2). No significant differences in functional connectivity were observed between groups for right aPFC (all *p* ≥ 0.22, two-tailed independent samples *t*-test corrected for multiple comparisons at the cluster level). Although aPFC connectivity was the main target of investigation in the current study, we also performed a supplementary seed-based functional connectivity analysis on other regions identified in ref. ^9^ to increase BOLD signal during lucid REM sleep, including left/right IPL, MTG and precuneus. The frequent lucid dream group showed increased connectivity between left IPL and left MTG, right lingual gyrus; right IPL and left aPFC, right PCC; right MTG and left aPFC, left MFG, and decreased connectivity between right IPL and right MFG, left insula, left precentral gyrus and left SMC (all *p* < 0.05, two-tailed independent samples *t*-test, corrected for multiple comparisons at the cluster level; Supplementary Table 1). No other suprathreshold clusters were identified.

**Table 2:** Whole-brain seed-based resting-state functional connectivity for left aPFC between groups.

	Volume (mm^3^)	*Peak t-value*	*p (cluster FDR)*	*Peak MNI*		
				*X*	*Y*	*Z*
*Frequent lucid dream group* > *Control group*						
L IPL (AG)	500	4.74	0.005	−36	−68	46
R IPL (AG)	671	4.56	0.001	48	−62	50
L MTG	258	4.25	0.04	−68	−24	−12
R MTG	262	4.08	0.04	64	−38	−6
R IFG	396	4.46	0.01	42	34	−14
*Frequent lucid dream group* < *Control group*						
L insula	1108	4.59	<0.001	−34	−20	6
R insula	696	4.40	0.001	32	−24	0

*Note*. IPL = Inferior parietal lobule; AG = angular gyrus; MTG = middle temporal gyrus, IFG = inferior frontal gyrus. All clusters significant at *p* < 0.05, cluster corrected. L: left, R: right.

### IPL/IPS subdivision analysis

**Figure 2:**
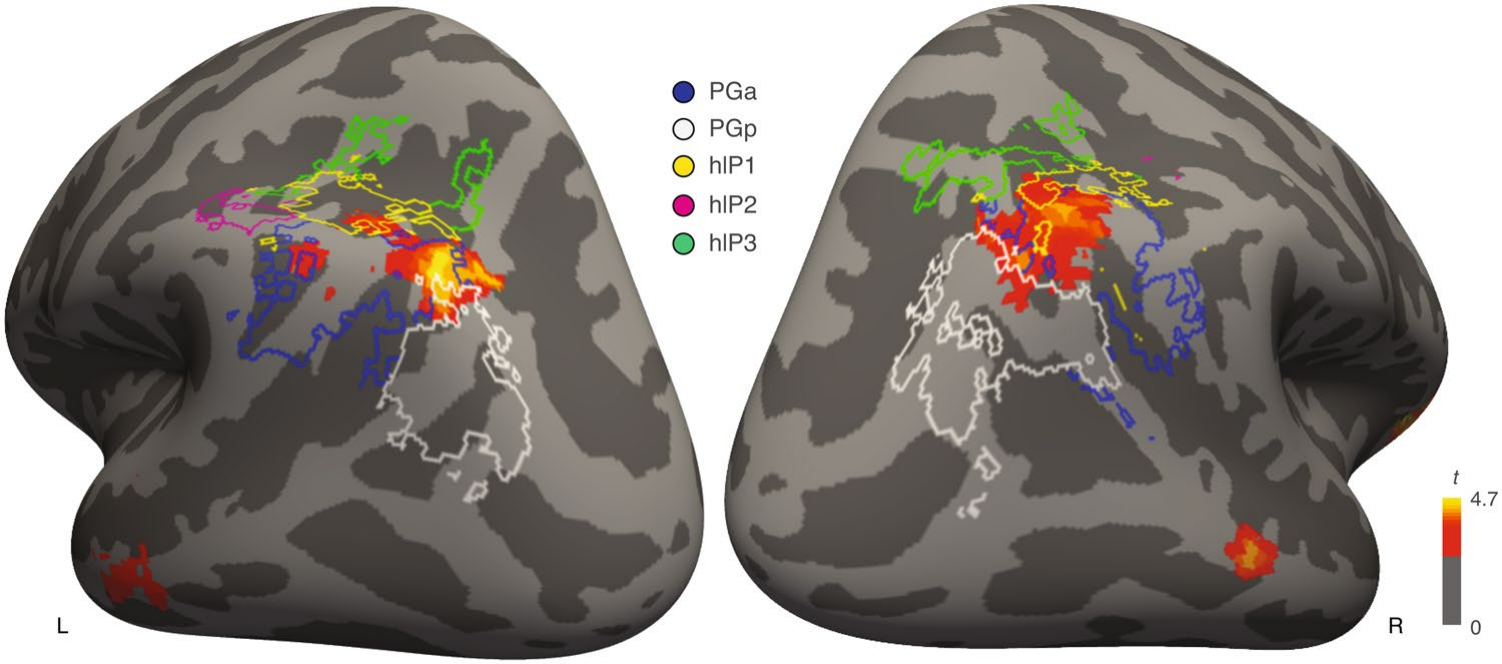
Clusters in lateral parietal cortex showing increased resting-state functional connectivity with aPFC in the frequent lucid dream group overlaid with cytoarchitectonic subdivisions of IPL/IPS. The angular gyrus can be subdivided into anterior (PGa; blue outline) and posterior (PGp; white outline) subdivisions based on cytoarchitecture. IPS can be divided into three subdivisions (hlP1 on the posterior lateral bank- yellow outline, hlP2 which is anterior to hIP1- purple outline, and hlP3 which is posterior and medial to both subdivisions- green outline). The cluster peak as well as maximal cluster extent localized bilaterally to a dorsal segment of the anterior angular gyrus (PGa). Region-of-interest (ROI) analysis revealed increased connectivity between left aPFC and bilateral PGa (blue outline) [all *p* < 0.05].

We performed a follow-up analysis on the clusters in left and right IPL in order to characterize the overlap between these clusters and anatomical subdivisions of the angular gyrus (PGa/PGp) and intra-parietal sulcus (hlP1, hlP2 and hlP3) (see *Methods: Angular gyrus (AG)/intra-parietal sulcus (IPS) subdivision analysis)*. The cluster peak for right parietal cortex was in the anterior AG (PGa) and the overlap between the functional cluster and the cytoarchitectonic maps was 47.3% for PGa, 24.7% for PGp, 4.2% for hlP1 and 0.6% for hlP3. The cluster peak for left parietal cortex was also in PGa and the overlap between the functional cluster and the cytoarchitectonic maps was 34.3% for PGa, 19.7% for PGp, 6.7% for hlP1 and 0.2% for hlP3 (Fig. 2). Frequent lucid dreamers showed significantly increased mean functional connectivity between left aPFC and left PGa (*t*(26)3.20, *p* = 0.004, two-tailed independent samples *t*-test), right PGa (*t*(26) = 2.46, *p* = 0.02, two-tailed independent samples *t*-test) and right hlP1 (*t*(26) = 2.59, *p* = 0.02, two-tailed independent samples *t*-test). No other anatomical subdivisions of AG/IPS showed significant differences between groups (all *p* ≥ 0.06, two-tailed independent samples *t*-test).

### Large-scale functional resting-state networks analysis

We next tested whether connectivity within and between established LSNs differed between groups. We first computed the average connectivity (Fisher-transformed correlation coefficients) within and between all pairs of nodes within 7 distinct systems identified in a meta-analysis^32^ (see *Methods: Large-scale networks analysis*). No significant differences in connectivity were observed between groups within any LSN (all *p* ≥ 0.29, two-tailed independent samples *t*-test) (Supplementary Fig. 1a). There were also no differences in between-network connectivity between groups (all *p* ≥ 0.16, two-tailed independent samples *t*-test). Next, we evaluated the overlap between our seed-based functional connectivity results and a 17-network parcellation of human brain connectivity^35^. The regions identified in our functional connectivity analysis overlapped with both default mode network (DMN) and frontoparietal control networks (FPCN), with the strongest overlap occurring within a subsystem of the FPCN (Supplementary Fig. 1b). We followed up this spatial overlap analysis by evaluating the connectivity within the FPCN subsystem that showed the largest overlap with the functional connectivity results, based on a 400 node parcellation of the 17 networks^36^. However, no significant difference in average network connectivity (average across all FPCN subsystem nodes) was observed within this network between groups (*t*(26) = −1.08, *p* = 0.29, two-tailed independent samples *t*-test). Thus, while the frequent lucid dream group showed increased functional connectivity of left aPFC with regions of IPL and MTG that overlapped with this FPCN subsystem, there was no difference in the average connectivity of this subsystem between groups.

### Whole-brain graph-theoretic analysis

**Figure 3:**
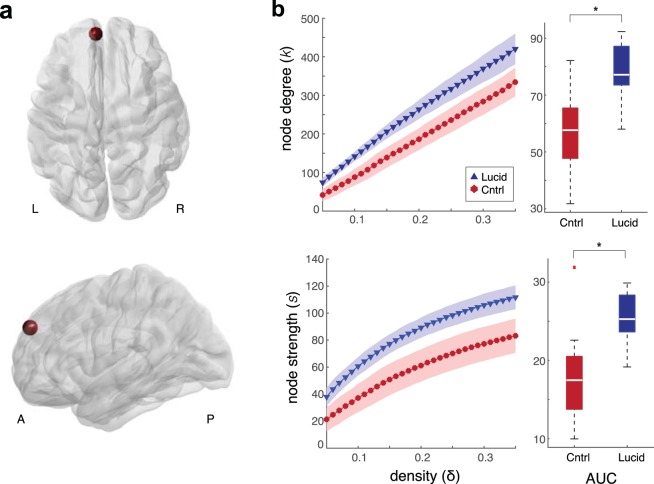
Whole-brain graph-theoretic network differences between frequent lucid dream and control groups. (**a**) aPFC node (red sphere) with significantly higher degree (*k*) and strength (*s*) in the frequent lucid dream group from axial (top panel) and left sagittal (bottom panel) views. (**b**) Left panel: Mean node degree (top row) and strength (bottom row) over density (cost factor) thresholds 0.05 ≤ δ ≤ 0.35 (step size 0.01) for frequent lucid dream (blue triangles) and control groups (red circles) for significant node shown in panel a. Shaded regions show 95% confidence intervals for each δ. Right panel: boxplots of area under the curve (AUC) for frequent lucid dream and control groups. The bottoms and tops of the boxes show the 25th and 75th percentiles (the lower and upper quartiles), respectively; the inner white band shows the median; and the whiskers show the most extreme data points not considered outliers (outliers are plotted separately with red squares). Asterisks indicate significant differences (*p* < 0.05) between conditions with a nonparametric bootstrap test after correcting for multiple comparisons against a surrogate max *t* distribution across all nodes.

To evaluate whole-brain differences in network and topological properties, we next parcellated the brain into 1015 regions according to the Lausanne 2008 atlas^37,38^ and performed graph-theoretic analysis. Graphs were thresholded over a range of connection densities (0.05 ≤ δ ≤ 0.35) for which the area under the curve (AUC) was computed for each node. Multiple comparisons were corrected against a max *t* distribution across all nodes in the network (see *Methods: Graph-theoretic network analysis*). Node degree and strength showed significant differences between groups in left aPFC after correcting for multiple comparisons, with higher node degree (*t*_*obs*_ = 4.58, *p*_*obs*_ = 0.0003, *p*_*corr*_ = 0.03, two-tailed independent samples *t*-test, max *t* corrected) and node strength (*t*_*obs*_ = 4.40, *p*_*obs*_ = 0.0003, *p*_*corr*_ = 0.04, two-tailed independent samples *t*-test, max *t* corrected) in the frequent lucid dream group compared to the control group (Fig. 3). No differences in betweenness centrality or eigenvector centrality were observed between groups for any node (all *p* > 0.05, two-tailed independent samples *t*-test, max *t* corrected).

## Discussion

### Summary of main findings

To the best of our knowledge, the current study is the first to evaluate differences in brain structure and functional connectivity of individuals who experience lucid dreams with high frequency. We found that compared to a control group matched on age, gender and dream recall frequency, individuals who reported lucid dreams spontaneously approximately every other night or more had increased resting-state functional connectivity between the left anterior prefrontal cortex (aPFC) and the bilateral angular gyrus (AG), bilateral middle temporal gyrus (MTG) and right inferior frontal gyrus (IFG). The frequent lucid dream group also showed decreased functional connectivity between left aPFC and bilateral insula. Whole-brain graph-theoretic analysis revealed that left aPFC had increased node degree and strength in the frequent lucid dream group compared to the control group. In contrast to these functional changes, we did not observe any differences in brain structure (gray matter density) in any brain area between groups (c.f. ref.^30^). Furthermore, no differences were observed between frequent lucid dream and control groups in behavioral or questionnaire measures of working memory capacity, prospective memory, mind-wandering frequency or trait mindfulness.

Our results converge with a recent fMRI case study of lucid dreaming, which found that a highly similar network of brain areas increased fMRI BOLD signal during lucid compared to baseline REM sleep, including bilateral aPFC, bilateral ITG/MTG, and bilateral medial/lateral parietal cortex (including AG)^9^. These same brain areas, particularly aPFC and IPL/AG, show reduced regional cerebral blood flow (rCBF)^13,14,39^ during REM sleep compared to waking (see ref.^15^ for a review). Hypoactivity of these regions coupled with preserved or increased activity in limbic/paralimbic structures and extrastriate cortices has been postulated to facilitate a mode of brain function conducive to hallucinatory dream mentation but diminished higher-order consciousness/self-awareness^40,41^. The current results suggest that increased functional integrity during wakefulness between aPFC and temporoparietal association areas—all regions that show suppressed activity in REM sleep and increased activity during lucid REM sleep—is associated with the tendency to have frequent lucid dreams.

### Lucid dreaming and brain connectivity

Becoming lucid during REM sleep dreaming involves making an accurate metacognitive judgment about the state of consciousness one is in, often by recognizing that the correct explanation for an anomaly in the dream is that one is dreaming^1,2^. The finding that changes in the functional connectivity of aPFC is associated with lucid dream frequency is therefore consistent with a large literature linking this region to metacognitive functions, including the evaluation of one’s thoughts and feelings^21,42^ and variance in the capacity to make accurate metacognitive judgments^23,24^.

Given the link to metacognition, it has been speculated that lucid dreaming is linked to neural systems that regulate executive control processes, in particular the frontoparietal control network (FPCN)^27,29^. The FPCN is a large-scale brain network that is interconnected with both the default mode network (DMN), which is linked to internal aspects of cognition, such as autobiographical memory^43,44^, spontaneous thought^45,46^, and self-referential processing^47^, and the dorsal attention network (DAN), which is involved in visuospatial perceptual attention^48,49^. Being spatially interposed between these two systems, the FPCN is postulated to integrate information coming from the opposing DMN and DAN systems by switching between competing internally and externally directed processes^49^.

Based on a parcellation of 17 resting-state networks in the human brain, which distinguished potentially separable FPCN networks^35^, a recent study found that the FPCN could be fractionated using hierarchical clustering and machine learning classification into two distinct subsystems: FPCNa, which is more strongly connected to the DMN than the DAN and is linked to introspective processes, and FPCNb, which is more strongly connected to the DAN than the DMN and is linked to regulation of perceptual attention^50^. The current results show that frequent lucid dreams are associated with increased functional connectivity between aPFC and a network of regions that showed substantial overlap with the FPCN sub-network corresponding most closely to FPCNa^35,50^. However, neither connectivity within FPCN broadly defined through meta-analysis nor connectivity within FPCN sub-networks as defined through parcellation of resting-state networks was significantly associated with frequent lucid dreaming in the current study. This may be attributed to both the partial overlap of the regions that showed increased aPFC connectivity in lucid dreamers with FPCN networks, as well as the fact that lucid dream frequency was associated with increased connectivity between these regions and aPFC in the left hemisphere, but not to increased connectivity between these regions and right aPFC, or broadly increased connectivity between other regions of FPCN to each other (outside of aPFC).

The strongest increase in functional connectivity in the frequent lucid dream group was observed between left aPFC and IPL, which localized to a dorsal segment of the anterior subdivision of the angular gyrus (PGa) bilaterally, as measured by overlap with cytoachitectonic probability maps. While many neuroimaging studies have treated the regions that comprise IPL as a homogenous region, cytoarchitectonic mapping studies have shown that these regions can be subdivided^51,52^, and these subdivisions show distinct patterns of structural and functional connectivity^53^. Specifically, PGa shows increased functional connectivity with the caudate, anterior cingulate, and bilateral frontal poles compared to PGp, whereas PGp shows increased connectivity with regions of the DMN, including precuneus, medial prefrontal cortex and parahippocampal and hippocampal gyri^53^. Cognitive or clinical correlates of altered functional connectivity between the frontal pole and this specific subdivision of AG (PGa) have to our knowledge not yet been identified, since much of the cognitive neuroscience literature on this region lacks anatomical specificity. However, a meta-analysis of 120 neuroimaging studies of language and semantic processes found that the left AG had the densest concentration of activation foci across studies, with a significant clustering of activation foci also in MTG^54^. The authors also note that these regions are greatly expanded in humans compared to non-human primates, suggesting a role in the development of language. Moreover, PGa is more closely linked to the semantic system that PGp, and analysis of the connectivity and cognitive functions associated with this region suggests that it is positioned at the top of a processing hierarchy for concept retrieval and conceptual integration^53^.

In line with these observations, we would like to offer a speculative hypothesis regarding our findings, which relates these results and the overlap with semantic/conceptual systems to the difference between lucid and non-lucid dreaming in terms of consciousness. Specifically, non-lucid dreams exhibit reduced working memory function, reduced ability to engage in behavioral control and planning, and reduced reflective consciousness^55–57^. Thus, while dreams are rich in primary consciousness of perception and emotion, consciousness during dreams typically lacks important aspects of what Edelman referred to as secondary or higher-order consciousness, which enables a creature to escape the “remembered present” of primary consciousness and to be conscious of being conscious^58,59^. In contrast, gaining lucidity during dreaming sleep involves regaining cognitive abilities associated with higher-order consciousness, including the ability to be explicitly aware of oneself and one’s state^55^. The distinction between primary and higher-order consciousness is thought to depend on the linguistic abilities that separate humans from other species^58^. While language processes also occur during non-lucid dreams^60,61^, they are nevertheless linked to the remembered present and apparently lack the conceptual structure that allows for full self-awareness. We speculatively propose that the aPFC-AG-MTG network identified here may be part of the neural circuitry enabling the integration between heteromodal metacognitive and linguistic/conceptual systems (in particular, the availability of AG-MTG semantic/conceptual content to anterior prefrontal regions) that allows one to be aware of oneself and one’s current state (i.e., “*I am dreaming!*”)^55^.

### Limitations, methodological considerations and future directions

The measurement of individual differences in lucid dream frequency has been done in inconsistent ways and could be improved in future research. In the current research we used a scale with a range of response categories, from “none” to “multiple times per night”^62^ (see *Supplementary Methods: Dream and lucid dream frequency questionnaire*). While this questionnaire provides a straightforward coarse assessment of lucid dream frequency, a limitation of this measure is that it does not measure variation in the length or “degree of awareness” of lucid dreams. Indeed, lucid dreams can range from a realization about the fact that one is dreaming followed by a loss of lucidity shortly thereafter to more extended lucid dreams in which an individual can maintain lucidity for prolonged periods of time^63^. Likewise, lucid dreams can be characterized by varying degrees of clarity of thought. Evaluating the duration of lucid dreams as well as the degree of awareness during lucid dreams will be valuable to relating brain structural and functional measures to lucid dream frequency in future studies. An extended discussion of this issue is beyond the scope of the present article; however, overall these remarks emphasize the need for the development of standardized measures that can be used to assess individual differences in frequency of lucid dreams that simultaneously measure the duration and degree of lucidity during dreams.

Another limitation of the current study is that our measurement of lucid dream frequency relied on questionnaire responses and participant interviews. There are established methods for the objective validation of individual lucid dreams in a sleep laboratory setting using volitional eye-movement signals^4^, but there are no protocols for physiologically validating the frequency of lucid dreams. While questionnaire measures of lucid dream frequency have shown high test-retest reliability^64^, one way to further validate participant questionnaire responses would be to attempt to physiologically validate at least one lucid dream in the sleep laboratory for each participant. We think that additional validations such as this would potentially be valuable to incorporate in future studies. Nevertheless, it is important to note that the estimated frequency of lucid dreaming would still depend on questionnaire assessment. Thus, approaches such as this do not obviate the reliance on questionnaire assessment as used in the current study. An intriguing, though ambitious, method for deriving a measure of lucid dream frequency not dependent on questionnaire assessment would be to utilize home-based EEG recording systems to collect longitudinal sleep polysomnography data, from which estimates of lucid dreaming frequency could be derived from the frequency of signal-verified lucid dreams collected over many nights. However, this approach would only measure the frequency of signal-verified lucid dreams, and instances in which participants achieved lucidity but did not make the eye signal due to factors such as awakening or forgetting the intention would be missed by this procedure.

In contrast to the observed differences in functional connectivity described above, in the current study we did not observe any significant differences in brain structure (gray matter density) between groups. This result contrasts with a study that found that two regions of aPFC had increased gray matter density in a “high-lucidity” group compared to a “low-lucidity” group^30^. As noted in the introduction, a limitation of that study is that the high-lucidity group was not a sample of frequent lucid dreamers, but rather individuals from a database that scored above the group median on a composite measure of dreaming, which measured not only frequency of lucid dreams but also different dimensions of dream content. While several of these content dimensions have been found to be higher in lucid dreams^57^, it is likely that several of these dimensions also co-vary more generally with dream recall and/or cognitive content in dreams unrelated to lucidity. As a consequence, as the authors note, some of the results could have been partly influenced by differences in dreaming “styles”, content or dream recall. However, the fact that the study found that these aPFC regions also showed increased BOLD activity during the monitoring component of a thought-monitoring task lends additional plausibility to the results. It is important to note that issues of statistical power could also account for the discrepant findings of these two studies. Unfortunately, no statistics or estimates of effect size have been reported for this effect and as a result we were unable to perform a power analysis to determine the adequate sample size for testing this effect. However, a single study that fails to reject the null hypothesis does not provide good evidence for the absence of an effect, especially with relatively small sample sizes. Overall, therefore, more research addressing this question using larger sample sizes will be needed before firm conclusions can be drawn.

Here we studied individuals who reported spontaneous lucid dreaming with high frequency without engaging in training to have lucid dreams. In our questionnaire samples, the proportion of individuals who reported spontaneous lucid dreams on close to a nightly basis constituted approximately 1 in 1,000 respondents. While frequent spontaneous lucid dreams are uncommon, evidence indicates that lucid dreaming is a learnable skill that can be developed by training in strategies such as metacognitive monitoring (i.e., “reality testing”) and, especially, prospective memory^65,66^. While it is plausible that the neurophysiological correlates of spontaneous frequent lucid dreaming are the same as frequent lucid dreaming that occurs as a result of training, this has not yet been studied. Future longitudinal training studies would be valuable in order to evaluate within-subject changes in brain connectivity as a result of training to have lucid dreams and to compare how such changes relate to the functional network associated with frequent lucid dreaming identified here.

No significant differences were observed between groups in working memory capacity, or questionnaire assessments of prospective memory or trait mindfulness. It has been suggested that a sufficient level of working memory is required in order to become lucid during dreaming sleep^2^ and thus it might be predicted that frequent lucid dreams could be associated with a higher baseline level of working memory capacity. Likewise, an effective method of lucid dream induction, the Mnemonic Induction of Lucid Dreams (MILD) technique^63^, relies on the use of prospective memory to become lucid, and thus it might be predicted that frequent lucid dreams could be associated with increased prospective memory ability. While we did not find evidence in support of a relationship between these variables and spontaneous frequent lucid dreams, it is worth noting that the relation between lucid dreaming and working memory has been discussed primarily in the context of successfully being able to “activate the pre-sleep intention to recognize that one is dreaming” during a dream^2^, and the relation to prospective memory is mostly considered in the context of learning to have lucid dreams by remembering to recognize that one is dreaming. However, spontaneous frequent lucid dreamers neither necessarily need to activate a pre-sleep intention nor use prospective memory to remember to recognize that they are dreaming; instead, their lucid dreams tend to occur spontaneously without engaging in specific methods for inducing them. Thus, it remains plausible that there could be a relationship between working memory and prospective memory and (successful) training in lucid dreaming despite a lack of a relationship between these variables and spontaneous frequent lucid dreams. In future work it would be interesting to explore whether individuals with higher baseline scores on these measures show increased propensity in successfully training to have lucid dreams, as well as to quantify the association between potential improvements in these skills and lucid dream frequency as a result of training. Finally, the finding that there was no significant difference in mindfulness in frequent lucid dreamers is consistent with other research, which has found that outside of meditators, there does not appear to be an association between trait mindfulness and lucid dream frequency in the facets of mindfulness studied here (decentering and curiosity)^34,67,68^.

In future work it would be intriguing to build on these findings to evaluate whether high frequency lucid dreamers show increased functional connectivity and/or higher metabolism or BOLD signal in these regions during REM sleep. If so, this would suggest that it may be possible to bias these networks toward increased metacognitive awareness of dreaming during REM sleep, for example through techniques to increase activation of these regions. Notably, a recent double blind, placebo-controlled study found that cholinergic enhancement with galantamine, an acetylcholinesterease inhibitor (AChEI), increased the frequency of lucid dreams in a dose-related manner when taken late in the sleep cycle and combined with training in the mental set for lucid dream induction^62^. While the relationship between cholinergic modulation and frontoparietal activation is complex and depends on the task context and population under study (see ref.^69^ for a review), pro-cholinergic drugs in general tend to increase frontoparietal activity in conditions in which these areas show low baseline activation, which is thought to reflect increased attentional-executive functions^69^. Given that frontoparietal activity is typically suppressed during REM sleep, an intriguing follow-up to these findings based on the current results would be to examine whether AChEIs, and galantamine in particular, may facilitate lucid dreaming through increasing activation within the network of fronto-temporo-parietal areas observed here.

In line with the above ideas, several studies have attempted to induce lucid dreams through electrical stimulation of the frontal cortex during REM sleep. One study tested whether transcranial direct current stimulation (tDCS) applied to the frontal cortex would increase lucid dreaming^70^. While tDCS resulted in a small numerical increase in self-ratings of the unreality of dream objects, it did not significantly increase the number of lucid dreams as rated by judges or confirmed through the eye-signaling method. Another study tested whether applying transcranial alternating current stimulation (tACS) in the low gamma band (25 Hz and 40 Hz) to frontal regions would induce lucid dreams^71^. While it was reported that lucid dreams could be induced with a high success rate (58% with 25 Hz stimulation and 77% with 40 Hz stimulation), there are concerns about how lucid dreams were defined. Specifically, lucid dreams were not dreams that participants self-reported as lucid, nor dreams that were objectively verified to be lucid through the eye-movement signaling method. Instead, dreams were inferred to be lucid based on higher scores to questionnaire items measuring the amount of insight or dissociation^57^. Given that dissociation (i.e. “seeing oneself from the outside” or a “3rd person perspective”) has never been considered a defining feature of lucid dreams (e.g., refs ^1,72,73^), it is controversial to classify dreams as lucid based on higher ratings of dissociation. Furthermore, mean ratings in the insight subscale increased from approximately 0.1–0.2 in the sham stimulation to 0.5–0.6 in the 25 Hz or 40 Hz stimulation conditions. However, the scale anchors ranged from 0 (strongly disagree) to 5 (strongly agree), indicating that, on average, in the 25 Hz and 40 Hz stimulation conditions, participants disagreed that their dreams had increased insight. In summary, it remains unclear whether electrical brain stimulation techniques could be effective for inducing lucid dreams (see refs^19,62^ for further discussion). Nevertheless, given the current findings, stimulation of aPFC and temporoparietal association areas appears to be a worthwhile direction for future research attempting to induce lucid dreaming. Future studies might consider testing a wider range of stimulation parameters, particularly applied to aPFC, as well as combining stimulation with training in the appropriate attentional set for lucid dream induction.

## Methods

### Participants

In total, 28 right-handed participants (18 females, age = 22.6 ± 5.4 (mean ± SD), range 18–34) participated in the study. Participants were recruited via mass emails sent to University of Wisconsin-Madison faculty, staff and students. The study was described broadly as a study on brain structure and dreaming. Exclusion criteria for all participants included pregnancy, severe mental illness or any contraindications for MRI (e.g., metal implants or pacemakers). To determine study eligibility, participants completed a questionnaire that measured their dream recall and lucid dreaming frequency (described below). For the frequent lucid dream group, we recruited individuals who reported a minimum of 3–4 lucid dreams per week, or approximately one lucid dream every other night without engaging in training to have lucid dreams. We recruited control participants who were 1-to-1 matched to participants in the frequent lucid dream group on age, gender and dream recall frequency variables but who reported lucid dreams never or rarely. Specifically, for each participant in the frequent lucid dream group, we recruited a matched control participant that was the same age (date of birth <12 months apart), the same gender, a similar level of dream recall (see below) and lucid dream frequency of 1 per year or less. Signed informed consent was obtained from all participants before the experiment, and ethical approval for the study was obtained from the University of Wisconsin–Madison Institutional Review Board. The study protocol was conducted in accordance with the Declaration of Helsinki.

### Individual differences in lucid dreaming and dream recall frequency

Participants completed a questionnaire that measured their dream recall and lucid dreaming frequency (*Supplementary Methods: Dream and lucid dream frequency questionnaire*). Dream recall was measured with a 15-pt scale ranging from 0 (never) to 15 (more than one dream per night). Lucid dream frequency was measured with a 15-pt scale ranging from 0 (no lucid dreams) to 15 (multiple lucid dreams per night). To help ensure clear understanding of the meaning of lucid dreaming, participants were provided with a written definition along with the scale as follows: “Lucid dreaming is a special sort of dream in which you know that you are dreaming while still in the dream. Typically, you tell yourself ‘I’m dreaming!’ or ‘This is a dream!’” (See Snyder & Gackenbach^12^ for the importance of providing a definition in the assessment of individual differences in lucid dreaming frequency). Participants were also provided with a short excerpt of a written report of a lucid dream (see *Supplementary Methods* for full text of the definition and example of lucid dreaming provided on the questionnaire measure).

Several additional checks were made to ensure that participants had a clear understanding of the meaning of lucid dreaming. First, participants were asked to provide a written example of one of their lucid dreams, including how they knew they were dreaming. Second, participants were interviewed by the experimenters before being enrolled in the study to ensure that they had a clear understanding of the meaning of lucid dreaming. During the interview participants described several recent lucid dreams and confirmed the frequency with which they experienced lucid dreams through follow-up questions. Only participants who demonstrated unambiguous understanding of lucidity and met the frequency criteria as confirmed by both written and oral responses were enrolled in the frequent lucid dream group. The frequent lucid dream group also reported several additional variables related to their experiences with lucid dreaming, including the number of lucid dreams they had in the last six months, the most lucid dreams they had ever had in a six-month period, whether they had engaged in training to have lucid dreams and their general interest in the topic.

As noted above, we aimed to match dream recall between the frequent lucid dream group and control group as closely as possible in order to control for this potentially confounding variable. However, it was not always possible to recruit a matched control participant that was exactly matched on age, gender and dream recall. For each participant in the frequent lucid dream group, we therefore sought to recruit the closet matched pair control participant of the same age and gender, with the constraint that dream recall had to be within at least 3 rank order values on the questionnaire measure. In 7 cases, we were able to obtain an exact match between control participants and frequent lucid dream participants on dream recall, in 5 cases within 1 rank value, in 1 case within 2 rank values and in 1 case within 3 rank values. In 4 out of the 5 cases that were within 1 rank value, the difference in reported dream recall frequency was between 7 dreams recalled per week and 5–6 dreams recalled per week, and in the remaining case the difference was between 3–4 dreams recalled per week and 5–6 dreams recalled per week. Overall this method ensured that the frequent lucid dream group and control group were closely matched on dream recall frequency.

### Behavioral and questionnaire assessment

Participants completed several additional assessments that measured cognitive variables which have been hypothesized to be associated with lucid dreaming and have been linked to PFC function, including working memory capacity (WMC), trait mindfulness and prospective memory (e.g., refs^2,33,34^). To measure WMC, participants completed automated versions of the operation span task (OSpan), rotation span task (RotSpan) and symmetry span task (SymSpan)^74^. These tasks have been validated to yield a reliable measure of WMC^75,76^. In brief, each task presents to-be-remembered stimuli in alternation with an unrelated processing task. In the OSpan the to-be-remembered stimuli are letters and the unrelated task is verifying the accuracy of an equation; in the SymSpan the to-be-remembered stimuli are locations of red squares in a 4 × 4 grid and the unrelated task is verifying the vertical symmetry of an image; in the RotSpan the to-be-remembered stimuli are arrows pointing in one of eight different directions and the unrelated task is whether a rotated letter is presented correctly. Participants completed two blocks of each task, which together provide a reliable measure of an individual’s WMC^75^. Following standard scoring procedures, span scores were calculated as the total number of items recalled in correct serial order across all trials^76^.

Participants also completed a questionnaire battery that assessed several additional variables of interest: their mind-wandering frequency, memory function in everyday life and trait mindfulness. Mind-wandering frequency was assessed with the Daydreaming Frequency subscale of the Imaginal Process Inventory (IPI)^77^. Memory function was assessed with the Prospective and Retrospective Memory Questionnaire (PRMQ)^78^, which measures self-report scores of the frequency of both prospective and retrospective memory errors in everyday life (see ref.^79^ for normative data). Trait mindfulness was measured with the Toronto Mindfulness Scale (TMS)^80^. The TMS measures two factor-analytically derived components of mindfulness: Curiosity and Decentering. The Curiosity factor corresponds to an “an attitude of wanting to learn more about one’s experiences”, whereas the Decentering factor corresponds to “awareness of one’s experience with some distance and dis-identification rather than being carried away by one’s thoughts and feelings”^80^.

### MRI acquisition

Resting-state functional MRI scans were collected on a 3.0 Tesla GE MRI scanner at the Wisconsin Institute for Sleep and Consciousness/HealthEmotions Research Institute (Department of Psychiatry) at the University of Wisconsin - Madison. A T2*-weighted echo-planar imaging (EPI) sequence was used (TR = 2000 ms; TE = 25 ms; flip angle = 60°; acquisition matrix = 64 × 64; FOV = 204 mm; acquisition voxel size = 3.75 × 3.75 × 4.00 mm; 40 interleaved slices, number of volumes = 300, duration = 10 minutes). During the resting-state scan, participants were instructed to stay awake and relax, to hold as still as possible, and to keep their eyes open. Before the functional scan, high-resolution T1-weighted anatomical scans were acquired (BRAVO, TR = 9180 ms; TE = 3.68 ms; TI = 600 ms; flip angle = 10°; FOV = 256 mm; acquisition voxel size = 1 × 1 × 1 mm).

### Structural (T1) data processing

T1 anatomical scans were segmented into gray matter (GM), white matter (WM), and cerebrospinal fluid (CSF) using SPM12 (Statistical Parametric Mapping, Wellcome Trust Centre for Neuroimaging, London). A diffeomorphic non-linear registration algorithm (diffeomorphic anatomical registration through exponentiated lie algebra; DARTEL)^81^ was used to iteratively register the images to their average. The resulting flow fields were combined with an affine spatial transformation to generate Montreal Neurological Institute (MNI) template spatially normalized and smoothed Jacobian-scaled gray matter images. Spatially normalized images were smoothed using an 8 mm full width at half maximum (FWHM) Gaussian kernel. We additionally evaluated average gray matter density between groups in the two regions of prefrontal cortex and bilateral hippocampus observed by ref.^30^ to show increases in a “high lucidity” group. We defined spherical ROIs of 4 mm radius in MNI152 space centered on the peak voxels reported in ref.^30^: right prefrontal (MNI: 4, 57, 31), left prefrontal (MNI: −30, 51, 6), left hippocampus (MNI: −21, 31, 3) and right hippocampus (MNI: 21, 31, 3). Total hippocampal volume was also extracted from an updated routine for automated segmentation of the hippocampal subfields implemented in FreeSurfer version 6.0^82^.

### Resting-state fMRI (EPI) data processing

Resting-state fMRI data were processed based on a workflow described previously^24^. To remove potential scanner instability effects, the first four volumes of each EPI sequence were removed. This was followed by slice timing and rigid-body motion correction to the mean EPI image in AFNI^83^. To compare head motion between groups, head motion was calculated by mean framewise displacement (FD) using Jenkinson’s relative root mean square (RMS) algorithm^84^. Affine transformation from mean EPI image to T1 volume was calculated using BBRegister^85^ and nonlinear transformation from T1 to the 2 mm MNI152 template was calculated using Advanced Normalization Tools (ANTs)^86^. Brain mask, cerebrospinal fluid (CSF) mask and white matter (WM) mask were parcellated using FreeSurfer^87–90^ and transformed into EPI space and eroded by 2 voxels in each direction to reduce partial volume effects. Realigned timeseries were masked using the brain mask. Differences in global mean intensity between functional sessions were removed by normalizing the mean of all voxels across each run to 100. Simultaneous surface and volume smoothing was applied using FreeSurfer: Cortical voxels were sampled to the surface and smoothed in surface space with a 10 mm FWHM Gaussian kernel while subcortical voxels were smoothed separately in volume space with a 5 mm FWHM Gaussian kernel. Outliers in the EPI sequence were discovered based on intensity and motion parameters using ArtDetect (http://www.nitrc.org/projects/artifact_detect). This was followed by nuisance regression of motion-related artifacts using a GLM with six rigid-body motion registration parameters and outlier scans as regressors. Principal components of physiological noise were estimated using the CompCor method^91^. Joined WM and CSF mask and voxels of highest variance were used to extract two sets of principal components (aCompCor and tCompCor). Timeseries were then denoised using a GLM model with 10 CompCor components as simultaneous nuisance regressors. Note that global signal regression was not performed because this processing step can induce negative correlations in group-level results^92^. Finally, timeseries data were temporally filtered (high-pass = 0.01 Hz, low-pass = 0.1 Hz).

### Seed-based whole-brain functional connectivity

To estimate connectivity, spherical regions of interest (ROIs) of 6 mm radius were defined in the MNI152 space (Fig. 1a) based on the peak voxel (MNI: −26, 62, 10; and homologous (x-flipped) coordinate) in aPFC reported in ref.^9^ to show increased BOLD signal during lucid compared to non-lucid REM sleep. In order to ensure that the spheres were contained within the pial surface of the cortex, spheres were shifted by two voxels in the x and y dimensions yielding a final MNI coordinate of x =  ± 24, y = 64, z = 10. Although aPFC functional connectivity was the main target of the current investigation, we also performed supplementary seed-based functional connectivity analysis on other regions identified in ref.^9^ to increase BOLD signal during lucid REM sleep, based on the peak voxel coordinates in left inferior parietal lobule (IPL) (MNI: −50, −52, 52), right IPL (MNI: 38, −62, 52), left inferior temporal gyrus/middle temporal gyrus (ITG/MTG) (MNI: −54, −60, −16), right ITG/MTG (MNI: 64, −38, −14), left precuneus (MNI: −10, −68, 42) and right precuneus (MNI: 8, −78, 48). ROI masks were transformed back to each subject EPI space using inverse nonlinear MNI152 to T1 transform and affine T1 to EPI (thresholded after interpolation at 0.5). Translated ROIs were restricted within the cortical ribbon mask. ROI timeseries were estimated by averaging voxels within each ROI. Full brain connectivity (correlation) maps were calculated using AFNI^83^. Connectivity maps were z-transformed using Fisher’s *r-*to-*z* transform and then spatially transformed into MNI152 space. Group-level analysis was conducted using the general linear model (GLM) framework implemented in SPM12 (Wellcome Trust Department of Imaging Neuroscience, University College London, UK). Voxelwise independent samples *t*-tests were performed between groups. Whole-brain analyses were conducted, correcting for multiple comparisons using topological FDR^93^ at the cluster level. Cluster forming threshold was set at *p* < 0.0075 and cluster size threshold was set at *p* < 0.05 (cluster corrected). Surface rendering was performed using FreeSurfer and Surf Ice (https://www.nitrc.org/projects/surfice/).

### Angular gyrus (AG)/intra-parietal sulcus (IPS) subdivision analysis

Cytoarchitectonic mapping studies have shown that AG can be divided into anterior (PGa) and posterior (PGp) subdivisions and IPS can be divided into three distinct subdivisions (hlP1 on the posterior lateral bank, hlP2 which is anterior to hIP1, and hlP3 which is posterior and medial to both subdivisions)^51,52^. The subdivisions of AG and IPS have been shown to have distinct structural and functional connectivity patterns^53^. We performed a follow-up analysis on the functional clusters identified in our seed based functional connectivity analysis in order to characterize the overlap between these clusters and the anatomical subdivisions of these regions. Five regions of interest (ROIs) were constructed using maximum probability maps (MPMs) with the atlas probability maps from the Anatomy Toolbox v1.8 in SPM^94^. MPMs create non-overlapping regions of interest from the inherently overlapping cytoarchitectonic probability maps^94,95^. The anatomical boundaries of these maps are described in detail in previous publications^51,52,95^. Mean connectivity values from each binarized mask were exacted using the MarsBar toolbox^96^.

### Large-scale networks (LSNs) analysis

In order to compare whether connectivity within and between established large scale resting-state brain networks showed differences between groups, we extracted timecourses from a set of 166 nodes from a meta-analysis by Power, *et al*.^32^ corresponding to 7 different systems: the default mode network (DMN; 58 nodes), the cingulo-opercular network (CO; 14 nodes), the frontoparietal control network (FPCN; 25 nodes), the salience network (SN; 18 nodes), the ventral attention network (VAN; 9 nodes), the dorsal attention network (DAN; 11 nodes) and the visual system (VIS; 31 nodes). For each network, we calculated the mean correlation between all nodes within the network (within-network connectivity) as well as the mean correlation between all nodes of a given network and all the nodes of each other network (between-network connectivity). Correlation values were z-transformed using Fisher’s *r-*to-*z* transform. We also evaluated the overlap between our seed-based functional connectivity results and a 17-network parcellation of human brain connectivity networks^35^. The 17-network parcellation in MNI space was down-sampled from 1 mm isotropic to 2 mm isotropic to match the space of the functional connectivity results and the spatial overlap of all functional connectivity clusters with each network was calculated as the percentage of significant (cluster corrected) voxels within each network. We followed up this network overlap analysis by evaluating the connectivity between all nodes within the frontoparietal control subsystem that showed the largest overlap with the functional connectivity results, based on a 400 node parcellation of the 17 functional networks^36^.

### Graph-theoretic network analysis

To construct functional networks for graph-theoretic analysis, anatomical scans were segmented using FreeSurfer and parcellated into 1015 regions according to the Lausanne 2008 atlas included in the connectome mapping toolkit^37,38^. Parcellation masks were transformed back to each subject EPI space using the BBRegister affine T1 to EPI transform. Voxel-level fMRI timeseries in each subject’s native space within each mask were averaged and correlated to all other regions, yielding an adjacency matrix A whose entries A_ij_ reflect the functional connectivity between region *i* and region *j* for each subject. Resting-state fMRI data pre-processing was identical to the procedures described above (see *Resting-state fMRI data processing*) with the exception that no spatial smoothing was applied, as spatial smoothing can distort network measures derived from average timeseries within parcellated regions (e.g., ref.^97^). All network metrics were computed in Matlab v 9.1 (The MathWorks Inc., Natick, MA, 2008) using the Brain Connectivity Toolbox^98^. For each node in the network we analyzed the degree (*k*), strength (*s*), betweenness centrality (BC) and eigenvector centrality (EC). These metrics are described in detail elsewhere (see refs^98,99^ for reviews). In brief, *k* quantifies the total number of connections of a node, while *s* quantifies the sum of the weights of all connections to a node. BC and EC are different measures of centrality of nodes: BC is the fraction of all shortest paths in the network that contain a given node and EC quantifies nodes connected to other densely connected nodes as having high centrality.

In order to compare network and topological properties between groups it is important to ensure that graphs contain the same number of edges^99^. This can be achieved by thresholding A by the connection density (δ), also known as cost factor, of the network, which is the number of existing connections over the total number of possible connections^100,101^. Following recommended practice^99^, rather than apply a single threshold to graphs, which would limit any findings to a single arbitrary connection density, we thresholded graphs over a range of connection densities (0.05 ≤ δ ≤ 0.35) in steps of 0.01. For all measures except node strength, for which we computed undirected weighted matrices, network metrics were calculated on binarized thresholded matrices for each value of δ by setting all connections ≥δ to 1 and all connections <δ to 0. In order to compare groups over the range of thresholds, we calculated the area under the curve (AUC) of the δ-thresholded data by integrating the curve over the specified density range for each graph metric, as has been applied in previous studies (e.g., refs^101,102^). To test the null hypothesis of no difference in AUC between groups, we used a nonparametric bootstrapping procedure in which we randomly reassigned groups with replacement 10,000 times and computed a bootstrapped *t*-value for each node. To correct for multiple comparisons, the maximum *t*-value across all nodes for each surrogate distribution was recorded to obtain a maximum *t* distribution and the level of statistical significance was set against the maximum distribution at α = 0.05. This statistical approach has been used in previous studies and allows for strong control over type I error^103,104^.

## Electronic supplementary material

Supplementary Information

## Data Availability

The data that support the findings of this study are available from the corresponding author on reasonable request.
