# Supplementary material for: Frequent lucid dreaming associated with increased functional connectivity between frontopolar cortex and temporoparietal association areas
Source: Sci Rep. 2018 Dec 12;8:17798. doi: 10.1038/s41598-018-36190-w (PMC6290891; doi:10.1038/s41598-018-36190-w)
Supplement: Supplementary file 1 — Supplementary Information [file 41598_2018_36190_MOESM1_ESM.pdf]

Supplementary Information

Frequent lucid dreaming associated with increased functional connectivity between frontopolar cortex and temporoparietal association areas

Benjamin Baird[1*], Anna Castelnovo[1,2], Olivia Gosseries[1,3] & Giulio Tononi[1]

[1] *Wisconsin Institute for Sleep and Consciousness*
Department of Psychiatry
University of Wisconsin – Madison, USA

[2] *Sleep and Epilepsy Center*
Neurocenter of Southern Switzerland,
Civic Hospital (EOC) of Lugano, Switzerland

[3] *Coma Science Group*
GIGA-Consciousness,
University of Liege, Liege, Belgium

Supplementary Figure 1

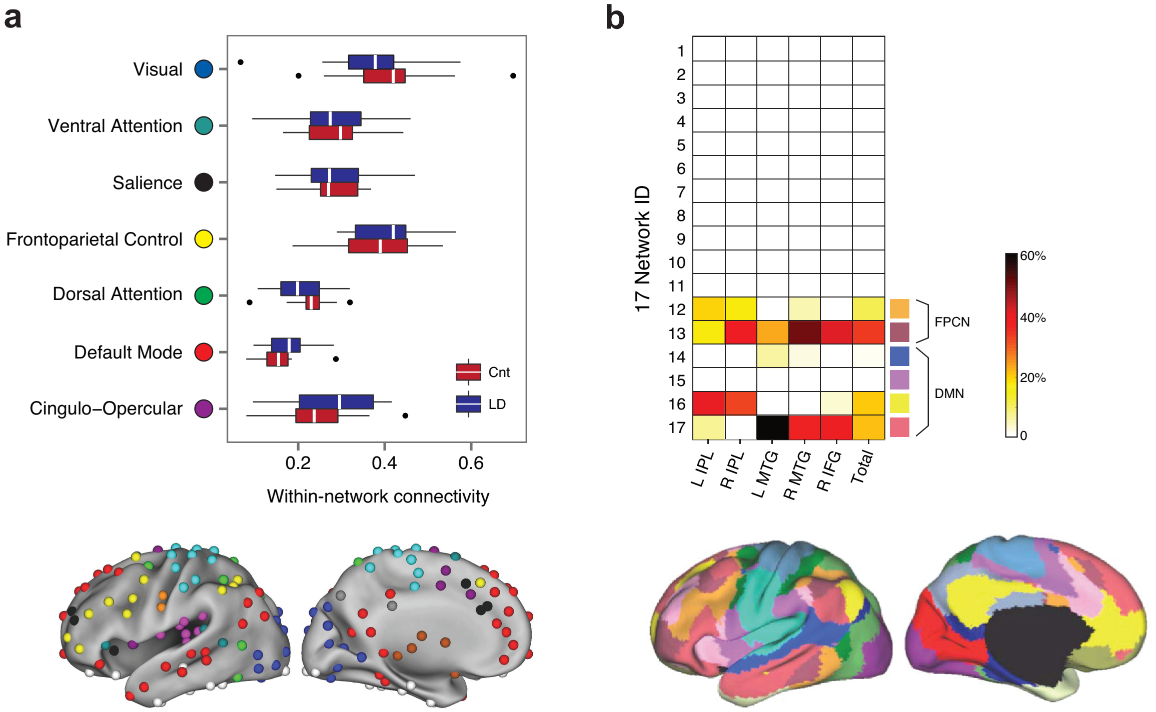

Fig. S1. (a) Average connectivity within large-scale networks (LSNs) between frequent lucid dream and control groups. No significant differences in resting-state functional connectivity were observed between groups within any LSN (all $p \geq 0.29$) as identified by meta-analysis. **(b) Overlap of resting-state functional connectivity results and a 17-network parcellation of resting-state functional networks.** Functional connectivity results overlapped with both default mode and frontoparietal control networks, with the strongest overlap occurring within a subsystem of the frontoparietal control network (network ID #13).

Supplementary Table 1

Table S1. Whole-brain seed-based functional connectivity for parietal and temporal regions between frequent lucid dream and control groups

Seed region	Clusters	Volume (mm^3)	*Peak t-value*	*p (cluster FDR)*	*Peak MNI*		
					X	*Y*	*Z*
**L IPL**	*Lucid dream group > Control group*						
	L MTG	424	5.25	0.008	-70	-24	-8
	R lingual	984	4.29	<0.001	18	-78	-2
	Lucid dream group < Control group						
	No suprathrehsold clusters	-	-	-			
**R IPL**	*Lucid dream group > Control group*						
	L aPFC	746	4.69	<0.001	-4	70	8
	R PCC	597	4.20	0.001	8	-30	34
	Lucid dream group < Control group						
	R MFG	775	5.09	<0.001	30	10	52
	L operculum/insula	319	3.68	0.02	-38	24	0
	L precentral/MFG	350	4.19	0.02	-38	0	44
	L SMC	323	3.94	0.02	-6	6	56
**L precuneus**	*Lucid dream group > Control group*						
	L/R lingual/fusiform/occipital	1230	4.02	<0.001	-24	-60	-2
	Lucid dream group < Control group						
	No suprathrehsold clusters	-	-	-			
**R precuneus**	*Lucid dream group > Control group*						
	No suprathrehsold clusters	-	-	-			
	Lucid dream group < Control group						
	No suprathrehsold clusters	-	-	-			
**L ITG/MTG**	*Lucid dream group > Control group*						
	No suprathrehsold clusters	-	-	-			
	Lucid dream group < Control group						
	No suprathrehsold clusters	-	-	-			
**R ITG/MTG**	*Lucid dream group > Control group*						
	L aPFC	330	4.59	0.03	-2	68	8
	L MFG	308	5.11	0.03	-34	14	60
	Lucid dream group < Control group						
	No suprathrehsold clusters	-	-	-			

Note. IPL=Inferior parietal lobule; MTG=middle temporal gyrus, ITG=inferior temporal gyrus; MFG=middle frontal gyrus; aPFC=anterior prefrontal cortex; SMC=supplementary motor cortex; PCC=posterior cingulate cortex. All clusters significant at $p < 0.05$, cluster corrected. L: left, R: right

Supplementary Methods: Dream and lucid dream frequency questionnaire

On the following pages are several statements/questions about your everyday experience with sleep and dreams. For each item please choose the alternative which is most appropriate or true for you. Please answer according to what really reflects your experience rather than what you think your experience should be.

1. Dream Recall
Approximately how often do you recall your dreams? (i.e., how often you wake from sleep and remember having a dream. You may—and usually do—forget them later.)

00 = Never
01 = Less than 1 dream per year
02 = 1 dream per year
03 = 2 dreams per year
04 = 3-5 dreams per year
05 = 6-8 dreams per year
06 = 9-11 dreams per year
07 = 1 dream per month
08 = 2 dreams per month
09 = 3 dreams per month
10 = 1 dream per week
11 = 2 dreams per week
12 = 3-4 dreams per week
13 = 5-6 dreams per week
14 = 1 dream per night
15 = More than 1 dream per night

2. Lucid Dream recall
Approximately how often do you recall lucid dreams (see Definition and Example)?

<u>Definition of Lucid Dreaming</u>: Lucid dreaming is a special sort of dream in which you know that you are dreaming while still in the dream. Typically, you tell yourself "I'm dreaming!" or "This is a dream!" This realization often (but not necessarily) leads to the ability to deliberately control one's actions or to observe passively the course of the dream with full conscious awareness, similar to the awareness you have while awake.

<u>Example of Lucid Dreaming</u>: "It was snowing gently. I was alone on the rooftop of the world, climbing K2. As I made my way upward through the steeply drifting snow, I was astonished to notice my arms were bare: I was wearing a short-sleeved shirt, hardly proper dress for climbing the second highest mountain in the world! I realized at once that the explanation was that I was dreaming! I was so delighted that I jumped off the mountain and began to fly away..."

00 = No lucid dreams yet
01 = Less than 1 LD per year
02 = 1 lucid dream per year
03 = 2 lucid dreams per year
04 = 3-5 lucid dreams per year
05 = 6-8 lucid dreams per year
06 = 9-11 lucid dreams per year

07 = 1 lucid dream per month
08 = 2 lucid dreams per month
09 = 3 lucid dreams per month
10 = 1 lucid dream per week
11 = 2 lucid dreams per week
12 = 3-4 lucid dreams per week
13 = 5-6 lucid dreams per week
14 = 1 lucid dream per night
15 = More than 1 LD per night

**If you answered 1 or higher to Question 2, please complete the following additional questions:

3. If you have ever had a lucid dream, please give an example, *including how you knew that you were dreaming*:

4. Lucid dreams in last six months
How many lucid dreams have you had in the last six months? Please enter a single, specific number as opposed to a numerical range (e.g., 7; not 5-10).

5. Most lucid dreams in six-month period
What is the greatest number of lucid dreams you've had in any six-month period? Please enter a single, specific number as opposed to a numerical range (e.g., 7; not 5-10).

6. For about how long (in years and months) have you been having lucid dreams?

7. Training. Have you ever engaged in training to have lucid dreams?
Yes
No
If Yes, please describe:
